# Research trends and hotspots in gastric carcinoma associated exosome: a bibliometric analysis

**DOI:** 10.3389/fonc.2024.1457346

**Published:** 2024-12-05

**Authors:** Chunqiu Liu, Honglei Guo, Fangzhou Jin

**Affiliations:** ^1^ Integrated Traditional Chinese and Western Medicine Oncology Department, Tangshan People’s Hospital, Tangshan, Hebei, China; ^2^ Department of Chinese Medicine, Tianjin Medical University General Hospital, Tianjin, China; ^3^ Graduate School, Tianjin University of Traditional Chinese Medicine, Tianjin, China

**Keywords:** exosome, gastric cancer, bibliometric analysis, biomarkers, tumor suppressor

## Abstract

**Background:**

Stomach cancer is considered the fifth most common cancer worldwide. This study utilized bibliometric analysis to construct a visualization map of the relationship between stomach cancer and exosomes, aiming to reveal research trends and emerging themes, and provide direction for future research.

**Method:**

Retrieve relevant literature on gastric cancer exosomes in the Web of Science Core Collection (WoSCC) over the past 25 years according to search criteria, and conduct bibliometric and visualization analysis using bibliometric software VOSviewer and CiteSpace.

**Results:**

This study included a total of 727 articles, with an overall increasing trend in annual publication output. There were 68 countries involved, with China having the largest number of publications followed by the United States. A total of 957 research institutions were involved, with most of the top 10 institutions in terms of publication output being universities in China. The top 5 journals are Molecular Cancer, Cell death & disease, Cancers, International journal of molecular sciences, and Frontiers in oncology. A total of 4529 authors were involved, with 5 authors having a publication output of no less than 13 articles. A total of 35516 references were cited, with a total number of citations. The top publication is “Exosome-mediated transfer of mRNAs and microRNAs is a novel mechanism of genetic exchange between cells”.

**Conclusion:**

Over the past 25 years, researchers have been dedicated to studying the field of exosomes related to gastric cancer, and research in this area is currently progressing steadily. Based on previous studies, exosomes in gastric adenocarcinoma serve as biomarkers, potential therapeutic targets, and post-resistance treatment, which represents current hotspots and emerging frontiers in research.

## 1 Introduction

Gastric cancer is a significant cancer worldwide, with over 1 million new cases in 2020 and approximately 769,000 deaths. It ranks fifth in global incidence and fourth in mortality, with men having twice the incidence rate compared to women (1). Although the combination therapy of surgery, chemotherapy, radiotherapy, and immunotherapy has improved the survival rate of these patients, most patients still experience recurrence and metastasis, leading to a poor prognosis. Research on the tumor microenvironment (TME) is a new approach in recent years to understand the pathogenesis of tumors and explore treatment methods (2). Among all components of the TME, extracellular vesicles play a crucial role.

Exosomes are disc-shaped vesicles with a diameter of 40-100nm that contain complex RNA and proteins, and are involved in intercellular communication (3). They mainly originate from multivesicular bodies formed by inward budding of the endosomal membrane, and are released into the extracellular matrix after fusion of the multivesicular body membrane with the cell membrane (4). In 1986, Johnstone and colleagues observed and harvested exosomes during the culture of sheep reticulocytes. However, because they lacked exosomal structure and biological activity, they were considered as ‘garbage’ produced by shedding of specific membrane functions (5). In 1996, Raposo et al. found that exosomes play an important role in antigen presentation *in vivo* (6). In 2007, Valadi et al. reported that exosomes from human and mouse mast cells contain abundant mRNA and microRNA that can be transferred to other cells, and the transferred exosomal mRNA can be translated in recipient cells, facilitating intercellular communication (7). Exosomes are naturally present in body fluids, and the precise molecular mechanisms underlying their secretion, uptake, composition, cargo, and corresponding functions have become a hot topic in medical research in recent years.

Tumor cells communicate with stromal cells by producing extracellular vesicles containing bioactive factors such as DNA, lipids, and ncRNAs, creating a favorable tumor microenvironment for tumor cell invasion and migration (8, 9). Helicobacter pylori infection is an important risk factor for the development of gastric cancer (10). Recent studies have found that extracellular vesicles play an important role in the connection between Helicobacter pylori infection and tumor development. Shimoda et al. reported that the virulence factor CagA of Helicobacter pylori can enter gastric epithelial cells via extracellular vesicles as nanocarriers, inducing these cells to adopt an elongated cell shape (11). In addition, Helicobacter pylori infection increases the expression of mesenchymal-epithelial transition factor (MET) protein in macrophages to promote the acquisition of a tumorigenesis-promoting phenotype and facilitate gastric cancer progression (12). These results suggest that extracellular vesicle-mediated molecular communication plays a crucial role in the crosstalk between Helicobacter pylori infection and gastric cancer. Exosomal miR-15b-3p promotes the migration and invasion of GC cells by targeting DYNLT1, caspase-3, and caspase-9 (13). Yang et al. found that miR-130a delivered by SGC exosomes promotes angiogenesis in GC cells by targeting c-MYB (14). It can be seen that extracellular vesicles are a promising research direction, with important implications for the study of the pathological mechanisms of gastric cancer, improving diagnostic accuracy, and exploring new treatment methods.

In the past decade, research in this field has shown an upward trend, with an increasing number of publications emerging. However, there is currently no study that analyzes the research trends and hot topics in the field of gastric cancer extracellular vesicles from a bibliometric perspective. To fill this gap, we used authoritative bibliometric tools such as VOSviewer and Citespace to analyze the main themes and citation processes of the well-studied topics in this field over the past 10 years, helping researchers identify hot topics that can be explored and those that have not yet been studied.

## 2 Method

### 2.1 Database

The Web of Science Core Collection is considered one of the most comprehensive databases in the field of scientific research, covering a wide range of interdisciplinary literature and research. It is one of the most suitable databases for accessing data (15). Therefore, we conducted a thorough literature search using the Web of Science Core Collection.

### 2.2 Search strategies

**Figure 1:**
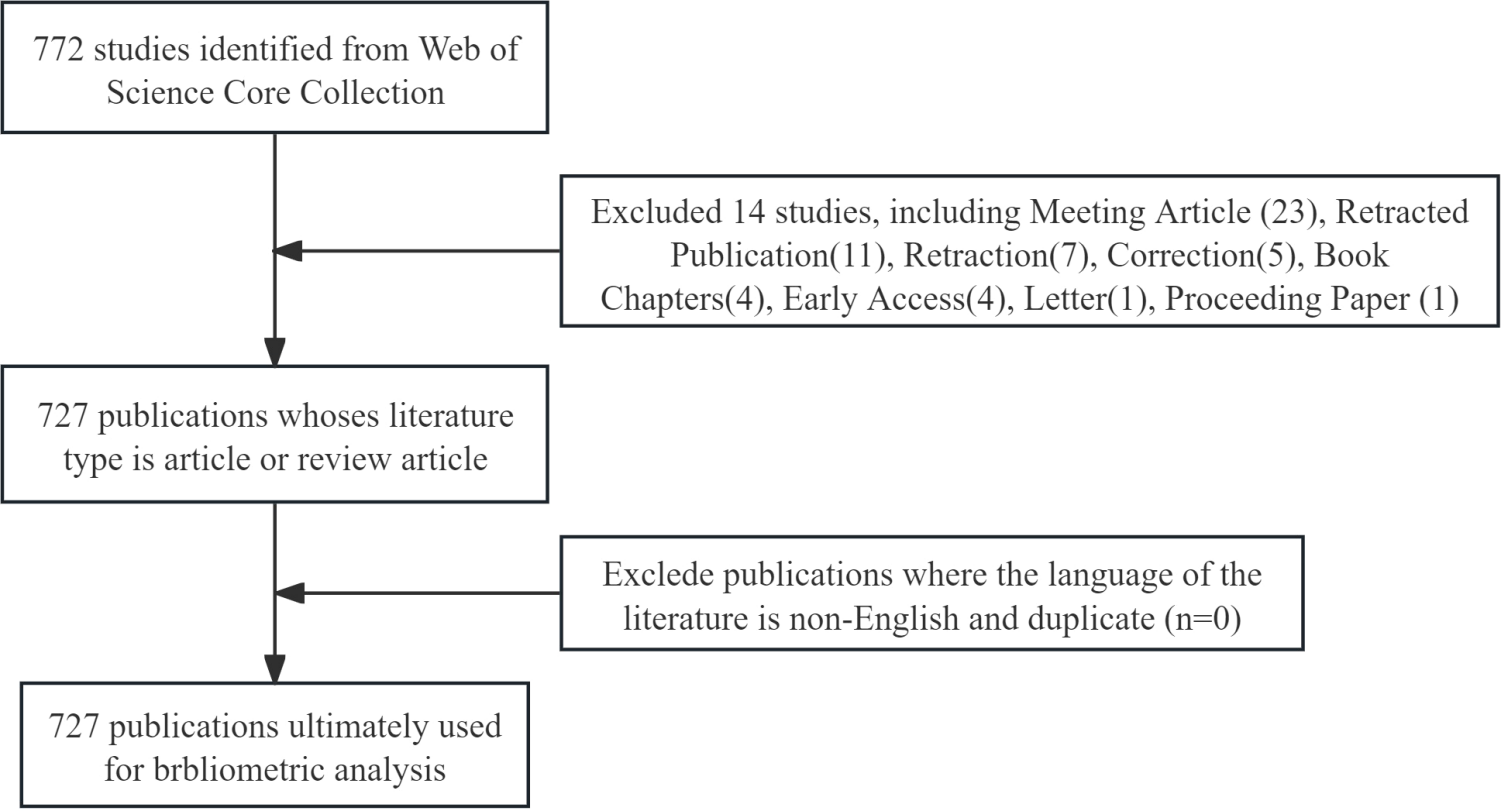
Publications screening flowchart.

Our search strategy was as follows:((TS=(gastric OR stomachic) AND TS=(cancer OR carcinoma OR tumor)) AND TS=(Exosomes OR Exosome)) AND LA = (English). The search was limited to 727 articles and review articles. Literature from April 15, 2009 to April 15, 2024 was selected. All 727 information of the retrieved literature was saved in plain text format for analysis (**Figure 1**).

### 2.3 Data visualization

In this study, we used a variety of tools for bibliometric analysis and visualization. VOSviewer is a widely used bibliometric analysis software that facilitates the visualization of relationships between different nodes and displays relevant information through their characteristics (16). In our study, we used VOSviewer to generate visualizations of co-author relationships of countries, institutions, authors, journals, citation relationships of references, and co-occurrence of keywords, providing concise and clear visual presentations. CiteSpace is a Java-based bibliometric analysis software that has unique advantages in burst detection, centrality calculation, citation relationship visualization, and cluster analysis (17). Therefore, we used CiteSpace to visualize dual-map of journals, burst of keywords or references, and co-citation relationships over time. We also utilized an online bibliometric analysis platform(bibliometric.com) to display the number of articles published each year, the number of articles published by country, and the distribution of keywords.

## 3 Results

We have conducted a search on the WoSCC database for articles and data related to exosomes in gastric cancer, utilizing the search formula, and ultimately collected 765 publications published between 2010 and 2022. Under the constraints imposed, we selected 727 publications for subsequent analysis (**Figure 1**).

### 3.1 Global trends in publication volume

**Figure 2:**
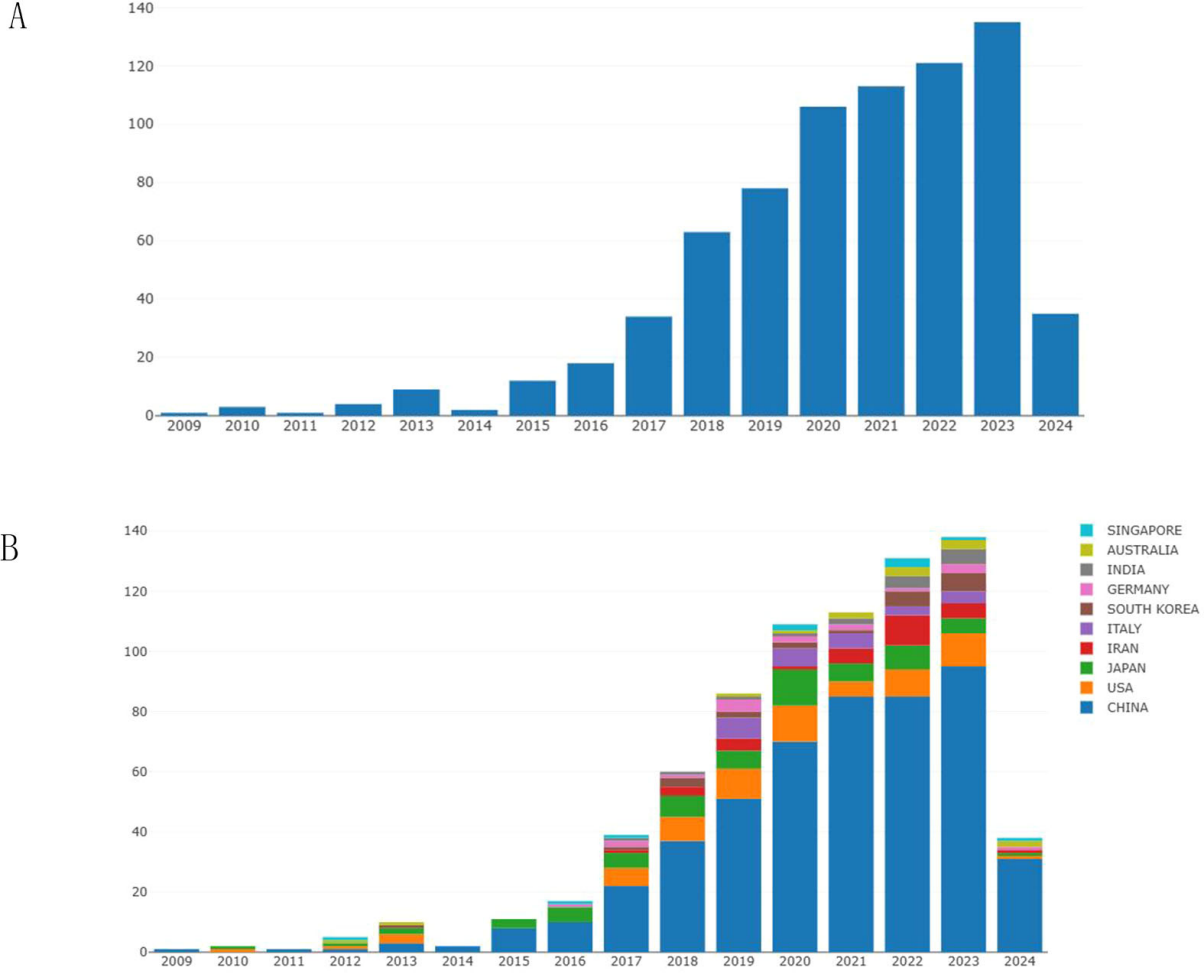
The annual publication volume in the field of exosomes related to gastric cancer. **(A)** Changes in the number of articles over the years. **(B)** The annual publication trends of the top 10 contributing countries/regions in this field.

We have collected and analyzed 727 publications on exosomes in gastric cancer over the past 25 years. **Figure 2A** shows the trends in global publication output and citation volume. Prior to 2017, the annual number of publications in this field did not exceed 40. After 2017, the number of publications in this field gradually increased, and in 2020, the number of publications exceeded 100 for the first time. Over the past 4 years, the average annual publication output has remained above 100 and continues to show an overall upward trend (**Figure 2A**). Examining the quantity of research papers and their changes over different periods helps reflect the popularity and development trends of exosome research in the field of gastric cancer.

**Figure 2B** shows the number of publications by country over the years. It can be observed that China has the highest number of publications, followed closely by the United States and Japan. Since 2015, the number of publications from China has exceeded the total number of publications from other countries, indicating that Chinese researchers have conducted more in-depth research in the field of gastric cancer exosomes.

### 3.2 Country analysis

We conducted a visualization study on publications related to gastric cancer exosomes. This study involved 62 countries. In **Supplementary Table 1**, we summarized the top 5 countries with the highest publication numbers and provided relevant information. China ranked first with a total of 495 publications, followed by the United States (65) and Japan (62). In terms of average citation rate per paper, the United States had the highest citation rate (57.45), followed by Singapore (44.00) and China (40.37). It is worth noting that Singapore has a relatively low publication output but a high citation rate, indicating that research findings from Singaporean researchers in the field of gastric cancer exosomes are worth referencing.

**Figure 3:**
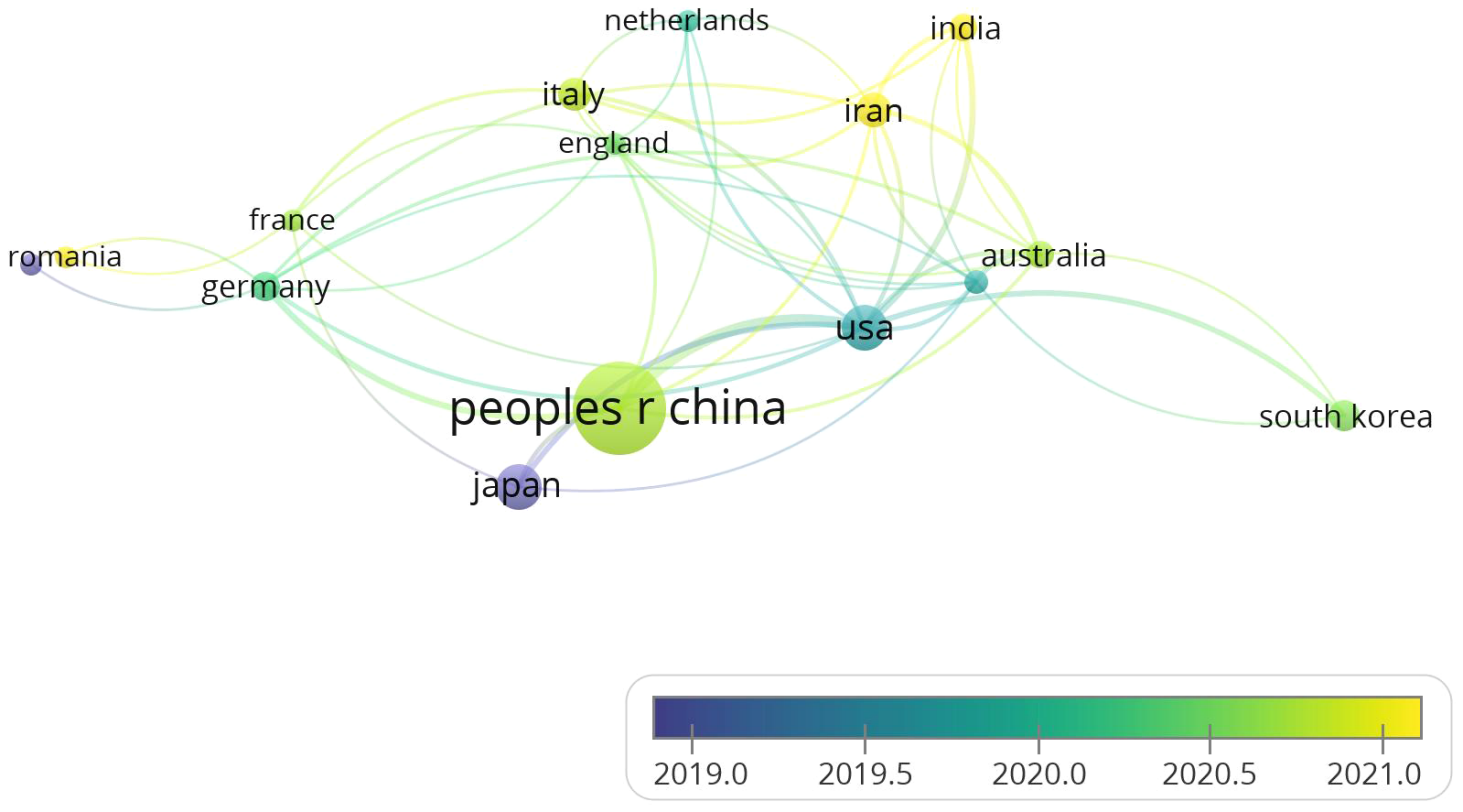
Visualizes the collaborative connection between countries.

All countries in this field cooperate with each other extensively and have complex connections. We use VOSviewer to analyze and visualize the collaboration among countries. As shown in **Figure 3**, China occupies a central position and has the highest number of collaborations with other countries. This indicates that they have sufficient coordination in exploring this field. The collaboration frequency between the United States and Japan closely follows. The color of the nodes can represent the chronological sequence of when they started their research. It is easy to find that the nodes related to Japan, Romania, and the United States are purple and teal, indicating that these countries had achievements in this field before 2020. The yellow nodes represent the research status of various countries in recent years, with India and Iran being newcomers in this area of research. Although China started later in this field, its total publication output exceeds that of other countries. In recent years, China has a large number of researchers, and the country attaches great importance to the training of research personnel. The establishment of research projects and funds provides research funding and resource support for researchers. With the expansion of higher education in China, the demand for high-level papers by Chinese researchers has significantly increased. In recent years, China has encouraged academic exchanges with the international academic community, and by publishing papers in international journals, researchers have established connections with international peers, shared research results, and enhanced China’s academic reputation in the field. These factors may contribute to the high publication output of Chinese researchers.

### 3.3 Institutions and funding agencies analysis

**Figure 4:**
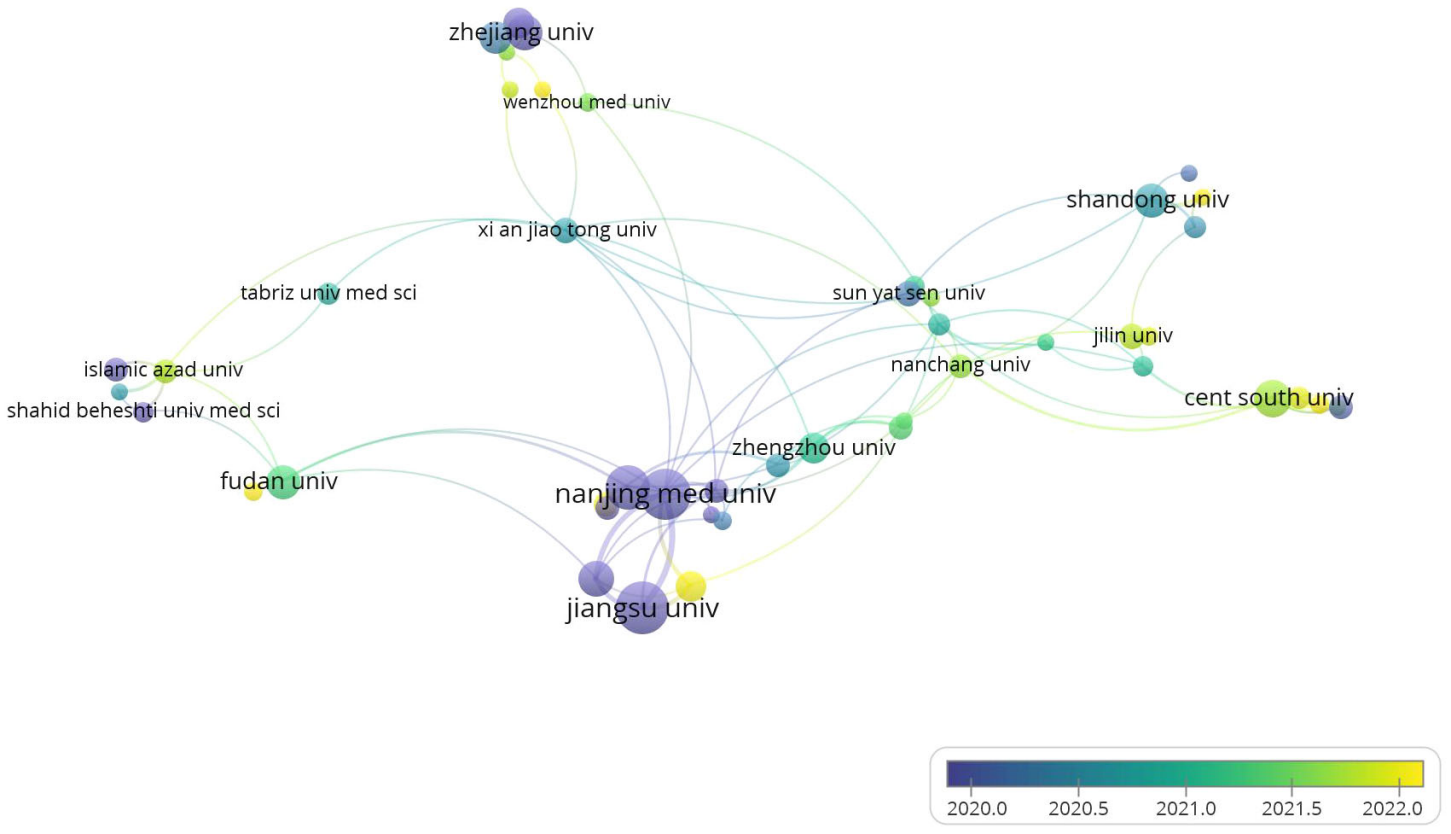
Visualizes the collaborative connection between institutions.

This study involved 957 institutions, with 52 appearing more than five times. As depicted in **Figure 4**, we analyzed the co-authorship patterns across various institutions. In **Supplementary Table 2**, we summarized the top 5 institutions with the highest total link strength and provided relevant information. Nanjing Medical University, Soochow University, Nanjing University and Nantong University, these five institutions have previously conducted research in this field. Nanjing Medical University and Soochow University, situated at the heart of collaborative authorship analysis. They boast the highest total link strength scores, respectively, of 31 and 21. **Figure 2** illustrates the timeline for each institution’s involvement in this field, with purple nodes indicating that these institutions began their research in this area at an earlier stage, such as Nanjing Medical University and Jiangsu University. Nantong University and Shandong University exhibit a yellow node, indicating that their research in the exosome field for gastric cancer began relatively late.

### 3.4 Journal analysis

**Figure 5:**
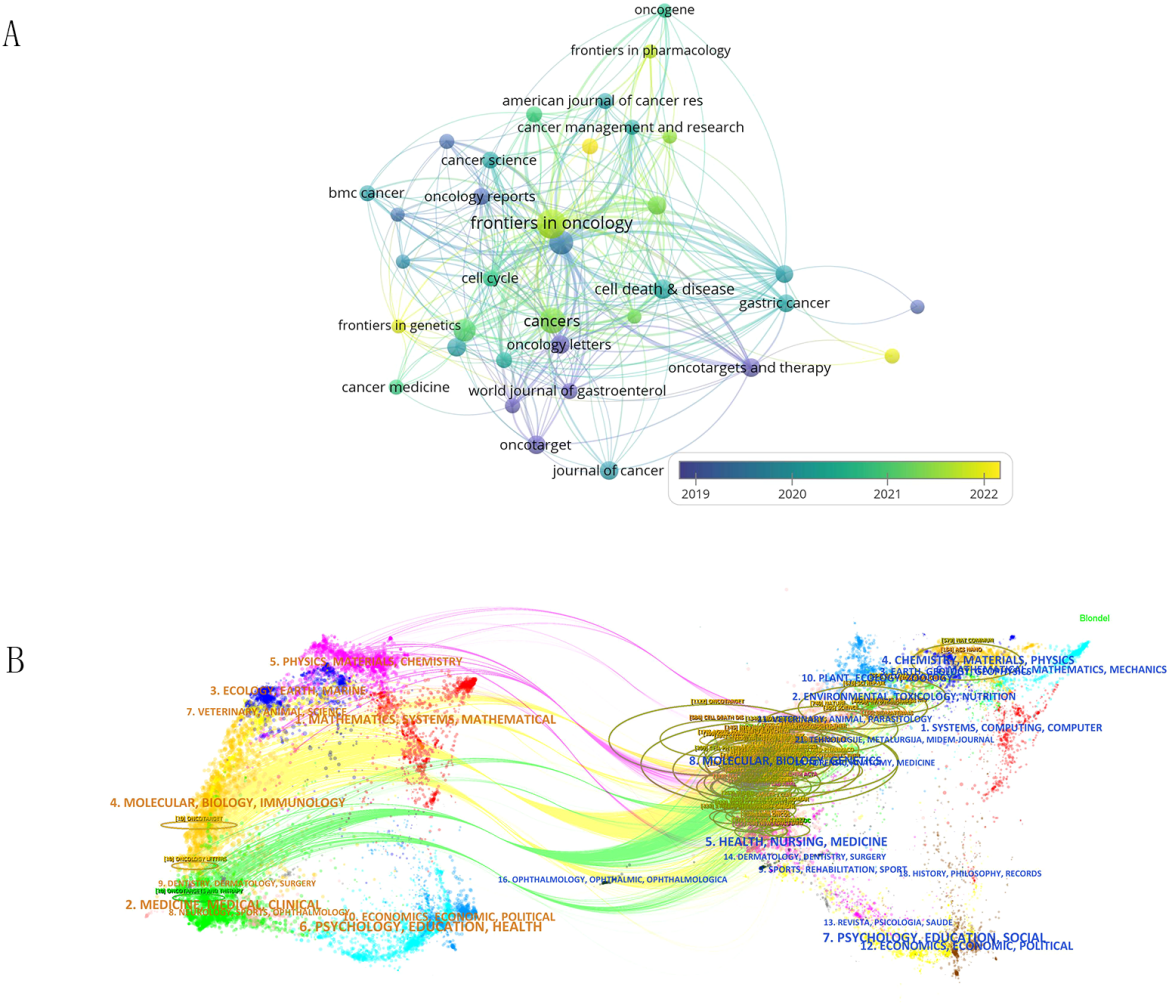
**(A)** Shows the citation relationships between journals. **(B)** Is the journal dual-map produced through CiteSpace.

In **Supplementary Table 3**, we summarize the top 5 journals with the highest number of publications and provide relevant information about these journals. The top 5 journals are Molecular Cancer, Cell death & disease, Cancers, International journal of molecular sciences, and Frontiers in oncology. **Figure 5A** shows the citation relationships among the 298 journals of all published literature in this field. The color of the nodes can reveal the time of publication of the papers, with purple and blue nodes representing publications before 2020, mainly related to research in the field by the American Journal of Cancer Research. The yellow nodes represent research after 2022, indicating emerging research from India and Iran in this area.

The dual-map overlay of journals shows the citation relationships between journals and co-cited journals, with clusters of citing journals on the left and clusters of cited journals on the right. As shown in **Figure 5B**, the green path is the main citation path.

### 3.5 Author and co-cited author analysis

**Figure 6:**
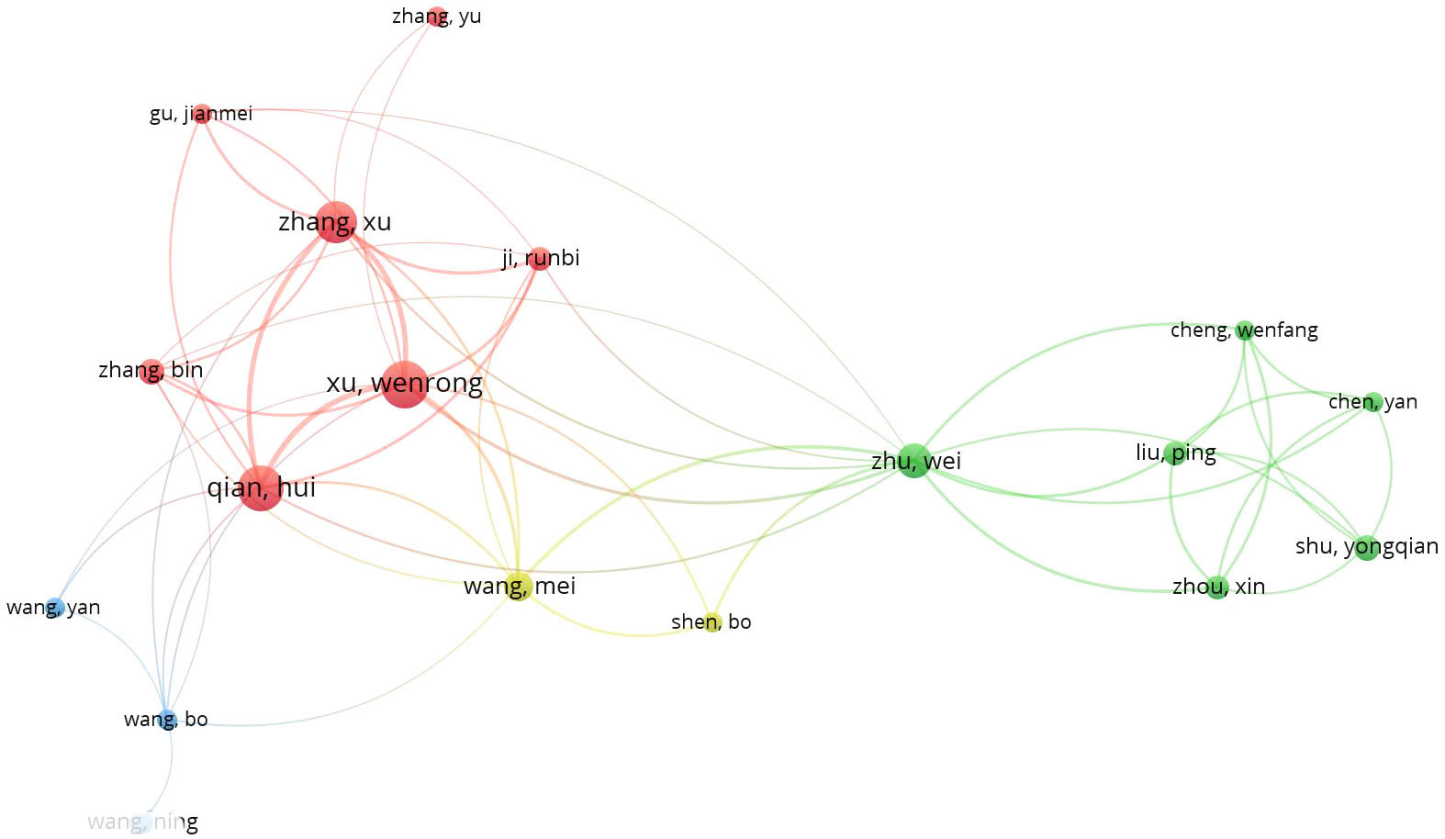
Visualizes the collaborations of the leading authors in the field.

Different researchers are conducting relevant studies in the field of gastric cancer exosomes. **Figure 6** shows the authors involved in research in this field since 2019, totaling 4529 individuals. Researchers were divided into groups represented by Xu wenrong, Wang mei, Zhu wei, and Wang bo, each making their own contributions in different areas related to gastric cancer exosomes. **Supplementary Table 4** displays information on the top 5 authors in terms of centrality. Among them, Ba yi, Zhang haiyang, and Deng ting have the highest centrality. The top 5 authors in this field have published 13 or more papers, with citation counts exceeding 1700, and an average citation rate higher than 110. Among them, Ba yi has the highest average citation rate per paper. He has the citation of 1981 and average citations per paper of 130 high. This indicates that the research of these authors has a profound impact on the advancement of gastric cancer exosomes field.

### 3.6 References and co-cited references analysis

**Figure 7:**
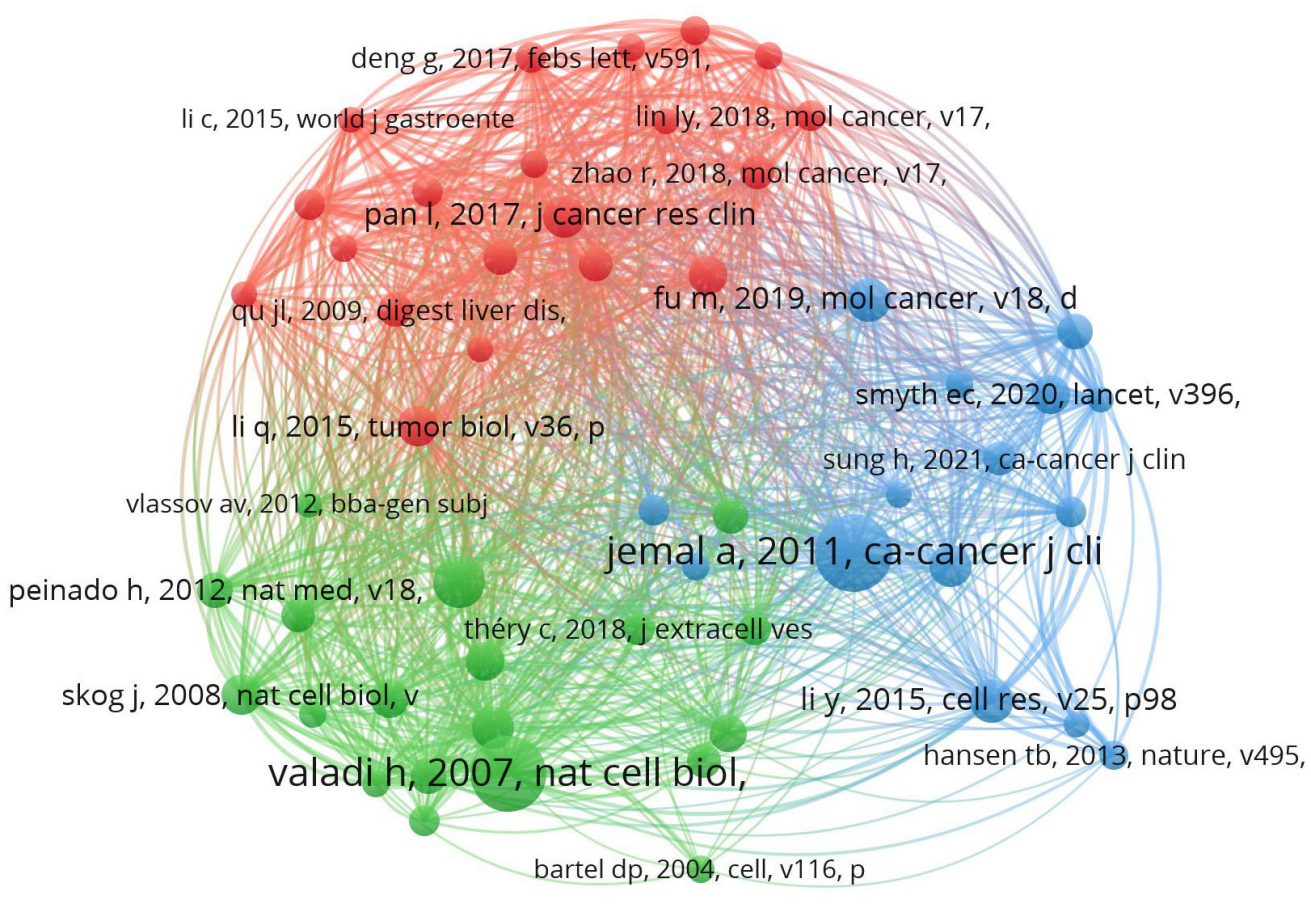
Visualizes the cluster analysis for references.

This study involves 35,516 co-cited references, with 126 of them cited more than 20 times. We used VosViewer for visualizing the references in our study (**Figure 7**). The references involved in this study can be mainly categorized into three types: reviews on extracellular vesicles, studies on molecular mechanisms related to extracellular vesicles, and studies on extracellular vesicles in the field of gastric cancer. **Supplementary Table 5** shows the information of the top 5 co-cited references in terms of centrality ranking. In 2007, Hadi Valadi discovered through cell culture experiments that ‘exosome-mediated transfer of mRNA and microRNAs is a new mechanism of genetic exchange between cells.’ Based on this finding, medical researchers conducted studies related to exosomes in the treatment of gastric cancer (7). In 2014, Qier Li (18) used quantitative reverse transcription polymerase chain reaction to analyze the plasma levels of LINC00152 in patients with gastric cancer, gastric dysplasia, and healthy controls. They also evaluated the levels of LINC00152 in plasma and exosomes using transmission electron microscopy, suggesting that exosome-protected plasma long non-coding RNA could serve as a potential stable biomarker for gastric cancer. In 2017, Lei Pan proposed that exosome-mediated transfer of the long non-coding RNA ZFAS1 promotes the progression of gastric cancer (19).

**Figure 8:**
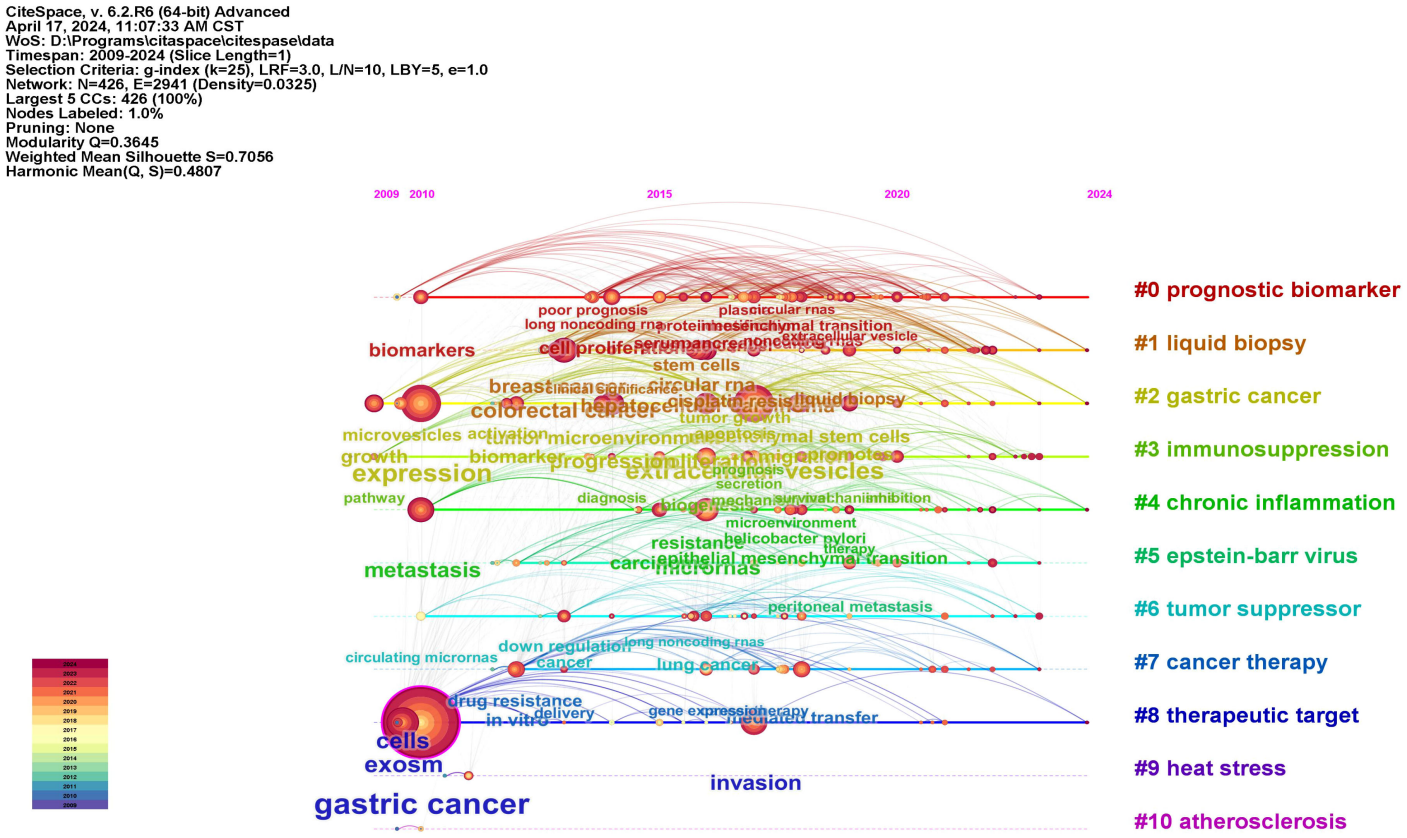
Timeline view map of reference co-citation analysis.

The co-citation analysis is an important feature of CiteSpace, typically used to identify popular research topics within a specific field. **Figure 8** provides a timeline view of the co-citation analysis of literature. Nodes within clusters represent common similarities in research focus, with each cluster represented by a ‘#’ tag extracted from the literature. Using the Log-Likelihood Ratio (LLR) algorithm, we divided all co-cited literature into 11 different clusters. Clusters with Q values exceeding 0.3 and S values exceeding 0.7 indicate the validity of the results. This is further supported by the use of the average publication year of the timeline or clusters, which helps in quickly assessing the evolutionary dynamics of each cluster.

### 3.7 Keywords analysis

**Figure 9:**
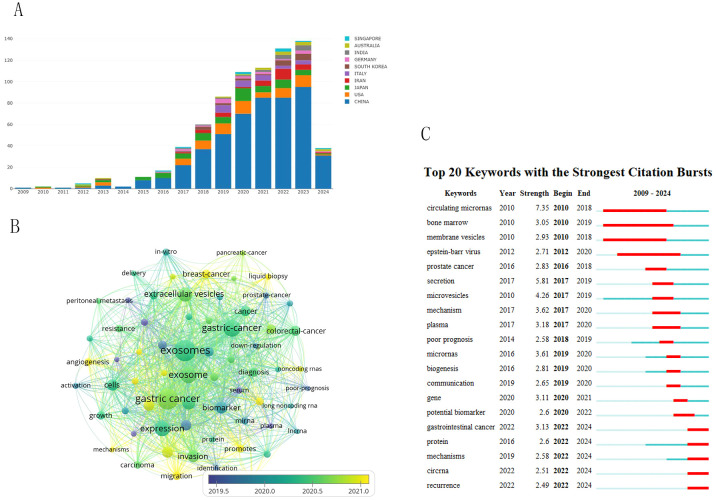
Visual analysis of keyword trends. **(A)** Changes in the number of key words over the years. **(B)** Visualizes the co-occurrence analysis of author keywords. **(C)** 7Shows the top 20 references with the strongest citation bursts.

**Figure 9A** shows the changes in key words in the field of gastric cancer exosomes over the years. This study began to focus on peritoneal metastasis, serum, and plasma, which are at the core of the keyword map. The yellow circles indicate recent hot academic topics, indicating that scientists are shifting their focus on liquid biopsy, angiogenesis, promotes, migration and mechanisms (**Figure 9B**). We used CiteSpace to detect keyword bursts and analyzed the top 20 strongest keywords. Early burst keywords included circulating micronas, bone marrow and membrane vesicles, strong bursts in circulating micronas and secretion, with strengths of 7.35 and 5.81, respectively. However, current bursts are seen in recurrence, circna, mechanisms, and gastrointestnal cancer (**Figure 9C**).

## 4 Discussion

In the rapid development of current science and technology, researchers face difficulties in choosing new research directions in many research fields. Mastering the current research trends is a necessary condition for researchers. Bibliometrics analysis is widely used to analyze the total number of papers in a field and the countries where they are published. Institutions, authors, and main figures can be identified, and their research trends can be predicted, providing researchers with a very convenient and fast way to understand a new field.The main role of exosomes is to transmit information between organic cells. They are more generally involved in the homeoenvironmental balance of cells and tissues by regulating cell viability, state, and function, and they can significantly mediate tissue repair. This study used bibliometric software to analyze the literature in the field of gastric cancer exosomes, conducting bibliometric and visualization analysis on basic information such as annual publication volume, authors, institutions, journals, countries, keywords, and citations.

The Nobel Prize in Physiology or Medicine in 2013 was awarded to scientists who made outstanding contributions in the field of cellular vesicle transport regulation, leading to subsequent research in the field of exosomes (20). As shown in **Figure 2**, before 2017, the annual publication output in the field of gastric cancer exosomes did not exceed 40 papers. After 2017, the number of publications in this field gradually increased, exceeding 100 papers for the first time in 2020. Over the past 4 years, the average annual publication output has remained above 100 papers, showing a continuing overall upward trend. This indicates that the field of gastric cancer exosomes is a relatively new research area, with explosive growth in research in this field after 2020. The incidence of gastric cancer is closely related to the Human Development Index (HDI) of a country. Compared to countries with medium or low HDI, countries with high HDI tend to have higher incidence rates of gastric cancer but relatively lower gastric cancer-related mortality rates (1). Countries such as Japan, Romania, and the United States started early in this field, which reflects their keen insight into research in emerging areas. Although China started later in this field, almost a year later than the United States, China’s contribution to this field is significant and cannot be ignored. As shown in **Figures 2B**, **3**, since 2015, China ranks first in total publications, citations, and TLS ranking in the field of gastric cancer exosomes. This is closely related to China’s leading global incidence rate and indicates that China has become a strong and influential country in gastric cancer exosome research.

We also studied the contributions of research institutions and funding agencies. The three institutions that were the earliest to conduct research in this field are Nanjing University, Jiangsu University, and Zhejiang University, all of which are leading institutions in the Chinese academic community. As shown in **Figure 4**, Nanjing University occupies a central position in the entire field. In addition, China has the most institutions with the highest number of publications, indicating significant influence in research in this field, deserving attention from scientists and institutions. When considering collaboration, priority should be given to these institutions.

For researchers, it is crucial to quickly find the right journals that contain cutting-edge publications in a certain field. We analyzed the journals and citations in the field of gastric cancer exosomes using VOSviewer and CiteSpace software. Molecular Cancer, Cell death & disease and Cancers urology stand out accounting for their high publications and citations.

Ba yi and Zhang haiyang, have the highest centrality (**Figure 6**), which is sufficient to illustrate their leadership roles in the field of exosomes and stomach cancer research. Professor Bayi comes from the Cancer Hospital of Tianjin Medical University, dedicated to the diagnosis and treatment of digestive system tumors and basic research. He has made outstanding contributions in the field of exosomes in gastric cancer. In 2020, the team led by Ba yi proposed that Cancer-associated fibroblasts-secreted miR-522 inhibits gastric cancer ferroptosis and promotes acquired drug resistance (21). This intercellular signaling reveals a new mechanism of acquired resistance in gastric cancer. In addition, Ba yi combined *in vitro* and *in vivo* experiments and found that GC cells derived exo-lncFERO controls GCSC tumorigenic properties through suppressing ferroptosis, and targeting exo-lncFERO/hnRNPA1/SCD1 axis combined with chemotherapy could be a promising CSC-based strategy for the treatment of GC (22). Chemoresistance is one of the reasons for the poor response of gastric cancer. Exosomes loaded with microRNAs (miRNAs), mRNAs, and other non-coding RNAs can regulate drug resistance. Ba yi found that Exo-anti-214 can reverse the resistance of gastric cancer to cisplatin, potentially serving as a promising alternative for the treatment of cisplatin-resistant gastric cancer in the future (23). Apatinib is currently used as third-line standard treatment for advanced gastric cancer, inhibiting tumor growth by targeting the anti-angiogenic pathway. Bayan discovered that exosomes secreted by gastric cancer cells carry miR-214-3p into vascular endothelial cells, directly targeting zinc finger protein A20, negatively regulating the iron efflux process key enzyme ACSL4, thereby inhibiting iron efflux in vascular endothelial cells and reducing the efficacy of Apatinib. Inhibiting miR-214-3p can increase the sensitivity of vascular endothelial cells to Apatinib, thereby promoting the anti-angiogenic effect of Apatinib (24). The above research shows that Professor Ba’s team has conducted extensive research on the treatment of gastric cancer, such as anti-angiogenesis, inhibition of chemotherapy resistance, using exosomes as a medium, and has achieved promising results.

Zhang haiyang found that hepatocyte growth factor (HGF) siRNA packaged in exosomes can be transported into cancer cells, significantly downregulating the expression of HGF to inhibit the proliferation and migration of cancer and vascular cells. In addition, exosomes can transfer HGFsiRNA *in vivo* to reduce the growth rate of tumors and blood vessels (25). This suggests that exosomes have the potential to deliver siRNA for targeted cancer therapy. Research by Zhang Ming has found that EGFR secreted by gastric cancer cells in exosomes can be transferred to the liver and integrated into the plasma membrane of hepatic stellate cells. Translocated EGFR can effectively activate HGF by inhibiting the expression of miR-26a/b. Therefore, Zhang Ming believes that exosomes containing EGFR from cancer cells may promote the development of liver-like microenvironment and facilitate liver-specific metastasis (26).

We conducted co-citation analysis, timeline analysis, and burst detection on the references. In recent years, the key focuses of gastric cancer exosomes can be summarized as the role of exosomes in gastric cancer drug delivery, the relationship between exosomes and anti-angiogenic drugs, the correlation between exosomes and gastric cancer metastasis and prognosis, and the relationship between exosomes and resistance to anti-tumor drugs.

With the advancement of medical technology, the use of biomarkers has become a new diagnostic option for early and rapid detection of GC. microRNAs, exosomes, circulating tumor cells, circular RNAs, cell-free DNA, and various proteins can serve as diagnostic biomarkers for GC patients (27). MiRNAs derived from exosomes may serve as more accurate cancer biomarkers than mRNA or proteins, aiding in the observation of tumor occurrence, prognosis, and responsiveness to treatment. Surgery is the main treatment method for gastric cancer. Therefore, early and rapid diagnosis of this malignant tumor is crucial for good treatment outcomes (28, 29). Through *in vitro* and *in vivo* experiments, Mengyan Xie found that the elevated expression of circSHKBP1 in gastric cancer tissues and serum is associated with advanced TNM stage and poor survival rate. The level of extracellular vesicle circSHKBP1 in patients after gastrectomy is significantly reduced. Overexpression of circSHKBP1 promotes the proliferation, migration, invasion, and angiogenesis of GC cells. Mechanistically, circSHKBP1 directly interacts with HSP90, blocking the interaction between STUB1 and HSP90, inhibiting the ubiquitination of HSP90, and accelerating the development of GC *in vitro* and *in vivo* (30). These results suggest that circSHKBP1 is a promising circulating biomarker for GC diagnosis and prognosis, as well as a specific candidate for further therapeutic exploration. Bin Xia’s research found that the serum exosomal LINC00691 in gastric cancer patients was significantly higher than that in healthy individuals and patients with benign gastric diseases. When GC exosomes were treated with normal fibroblasts (NFs), the level of LINC00691 increased significantly, leading to enhanced cell proliferation and migration, promoting the proliferation and invasion ability of GC cells. Knocking out LINC00691 or using the JAK2/STAT3 signaling pathway inhibitor ruxolitinib effectively deprived the conditioned medium of exosome-containing GC cells of its impact on NFs. This suggests that exosomal LINC00691 promoted NFs to gained the properties of cancer-associated fibroblasts (CAFs) depending on JAK2/STAT3 signaling pathway as a potential diagnostic biomarker for GC (31). Cuncan Deng found that the elevation of exosomal circATP8A1 is associated with advanced TNM stage and poor prognosis in gastric cancer patients. Experimental studies have shown that exosomal circATP8A1 derived from gastric cancer cells induces M2 polarization of macrophages and tumor progression through the activation of the circATP8A1/miR-1-3p/STAT6 axis. Knocking down circATP8A1 significantly inhibits the proliferation and invasion of gastric cancer. This suggests that circATP8A1 may serve as a potential prognostic biomarker and therapeutic target for gastric cancer (32). Has-circ-0000437 can promote the invasion, migration, and tube formation of human lymphatic endothelial cells (HLECs) *in vitro*, and can be enriched and transferred to HLECs through exosomes secreted by GC cells *in vivo*, promoting lymphatic vessel formation and lymph node metastasis (LNM) in a popliteal lymph node (PLN) metastasis model. These findings suggest that has-circ-0000437 may serve as a potential prognostic biomarker for gastric cancer patients with LNM, providing new targets for gastric cancer treatment (33). Guan Wang found that the expression of miR-619-5p in GC cells and their exosomes was proved to be significantly higher than that in normal cell lines. Nine key target genes of GC (BRCA1, RAD51, KIF11, ERCC6L, BRIP1, TIMELESS, CDC25A, CLSPN and NCAPG2) were identified, and a prognostic model was successfully constructed with a good predictive ability (34). Chen Xiaoming collected 595 cases of gastric cancer patients and analyzed the expression of CD63 in tumor cells and stromal cells using immunohistochemistry. It was found that CD63 was mainly expressed in the cell membrane of cancer cells and in the cytoplasm of stromal cells. Positive expression of CD63 was significantly associated with scirrhous gastric cancer, tumor depth, lymph node metastasis, lymphatic infiltration, and tumor size. The 5-year survival rate of CD63-positive tumor patients was significantly lower than that of CD63-negative tumor patients (p<0.001), suggesting that the expression of CD63 is an important independent factor for the prognosis of gastric cancer patients (35).

Although gastric cancer ranks fifth in global incidence, local lesions do not directly lead to patient death. However, the prognosis is usually poor when multiple metastases occur on the basis of the primary lesion of gastric cancer, which may be the main reason why gastric cancer ranks fifth in global cancer-related mortality. It can be inferred that metastasis in gastric cancer is the most deadly part of its disease progression. The intact mesothelium serves as a protective barrier against peritoneal metastasis. Guang Deng demonstrate that gastric cancer-derived exosomes promote peritoneal metastasis by inducing mesothelial barrier disruption and peritoneal fibrosis (36). Exosomes derived from peritoneal lavage in gastric cancer contain a large amount of microRNAs related to peritoneal metastasis. Studies have found that hsa-let-7g-3p and hsa-miR-10395-3p can serve as biomarkers for predicting peritoneal metastasis and systemic chemotherapy efficacy, and are involved in gastric cancer metastasis and chemotherapy resistance (37). Exosomes from gastric cancer cells, especially MKN-45 and MKN-28, alter the gene expression and cytokine secretion patterns of CD8+ T cells, creating an immune-suppressive environment for the formation of lung metastatic niche (38). Liver metastasis (LM) is a major obstacle affecting the prognosis of gastric cancer patients. Shengkui Qiu’s research found that exo-miR-519a-3p plays a critical role in mediating crosstalk between primary GC cells and liver macrophages, making it a potential therapeutic target for GC-LM (39).

Exosomes are widely present in various body fluids, including blood and urine, and can provide important information about the cells or tissues they originate from, which is crucial for assisting in the clinical treatment of diseases. Exosomes have high target specificity, the ability to cross cell membranes, immunotolerance, versatility, and feasibility for large-scale production. They can achieve precise targeted delivery through the interaction between the heterogeneous surface proteins of exosomes and matching proteins on target cells (40). Dan-Dan Shen ‘s research found that exosomes rely on the presence of LSD1 in donor cells *in vivo*, and can regulate the proliferation of Monocyte (MFC) cells through exosomal PD-L1-mediated T cell immune regulation. Knocking out LSD1 can reduce the expression of exosomal PD-L1 in GC, which helps restore T cell response. This finding provides a new target for immunotherapy of gastric cancer (41). CircGLIS3 is significantly upregulated in gastric cancer tissues, and its high expression is associated with advanced TNM stage and lymph node metastasis in gastric cancer patients. CircGLIS3 promotes proliferation, migration, and invasion of GC cells *in vitro* and *in vivo*, and can facilitate gastric cancer metastasis and polarization of macrophages towards the M2 phenotype. These findings suggest that CircGLIS3 may be a potential therapeutic target for gastric cancer patients (42).

Once tumors develop drug resistance, the drugs are no longer effective in fighting cancer. Due to the overactivation of drug efflux pumps, gene dysregulation, and interactions with the tumor microenvironment, tumor cells can develop resistance to Cisplatin (DDP) chemotherapy. One of the latest advances in overcoming DDP resistance in the treatment of gastric cancer is the application of nano-platforms, which can increase the accumulation of DDP at the tumor site and enhance its cytotoxic effects on cancer cells by inhibiting oncogenic factors and overcoming biological barriers (43). Oxaliplatin (OXA) is a first-line chemotherapy drug for gastric cancer. Xiao Ming found that exosomes isolated from M2-polarized macrophages (M2-Exos) can co-localize with gastric cancer cells. M2-Exos can reduce cell apoptosis and enhance OXA resistance. Circ0008253 can transfer from M2-Exos to gastric cancer cells. Overexpression of circ0008253 increases cell viability, tumor size, and ABCG2 levels, while reducing OXA sensitivity (44). The importance of exosomes released by cancer cells in the treatment of various cancers has been recognized by the public. Exosomes derived from GC cells transfer circ_0091741, inducing autophagy and OXA resistance in GC cells. circ_0091741 blocks the binding of miR-330-3p to TRIM14, increases TRIM14 expression, further activates the Wnt/β-catenin signaling pathway by stabilizing Dvl2, enhances gastric cancer cell autophagy and antioxidant capacity. The promoting role of exosomal circ_0091741 in gastric cancer cell autophagy and chemotherapy resistance lays the foundation for the development of new therapeutic targets for gastric cancer (45). Platinum resistance is a major cause of poor clinical prognosis in gastric cancer patients. However, the exact mechanism of platinum resistance remains unclear. Xinming Jing found that miR-769-5p delivered by exosomes promotes gastric cancer platinum resistance and progression by targeting CASP9 and promoting p53 ubiquitination and degradation. Targeting miR-769-5p with its antagonist can restore platinum responsiveness in resistant GC cells, confirming that exosomal miR-769-5p is a key regulatory factor in GC platinum resistance and can enhance the efficacy of anticancer chemotherapy, providing a new therapeutic option for gastric cancer (46). DACT3-AS1 is mainly transferred from cancer-associated fibroblasts (CAFs) to GC cells via exosomes. Downregulation of DACT3-AS1 expression is associated with poor prognosis in gastric cancer patients. DACT3-AS1 inhibits cell proliferation, migration, and invasion by mediating ferroptosis through the miR-181a-5p/SIRT1 axis, enhancing sensitivity of tumor cells to oxaliplatin (47).

Exosomes can be loaded with drugs, serving as drug carriers to deliver their contents, promote cell communication, facilitate the development and application of new drugs, and thereby formulate new treatment strategies. Chemotherapy drugs are essential for the treatment of gastric cancer, but they have problems such as high organ toxicity and unsatisfactory treatment effects. With the continuous advancement of medical technology, the development of nanomedicine delivery carriers with tumor-targeting functions and immune-stimulating capabilities can to some extent compensate for these shortcomings. iPSCs-DCs exosome fusion vector possesses both tumor-targeting and immune factor recruitment capabilities. Xiaoming constructed a nano drug delivery system, DOX@aiPS-DCexo, which effectively inhibits the expansion of gastric cancer MFC through synergistic chemotherapy and immunotherapy, significantly improving the *in vivo* therapeutic efficacy of chemotherapy drugs (48). TingGuo uses a multi-omics strategy, utilizing RNA sequencing technology, to characterize the mRNA, miRNAs, and lncRNAs profiles of circulating extracellular vesicle-enriched components from paired responders and non-responders to neoadjuvant chemotherapy (NACT) in AGC patients. They identified many miRNAs, mRNAs, and lncRNAs associated with the response of AGC patients to NACT and validated them through cohort studies. Based on this, they established a 6-exosome-RNA panel that could robustly identified responders from non-responders treated with fluorouracil-based neoadjuvant chemotherapy (49). Exosomes are first formed by invagination of the cell membrane, complete the packaging process inside the cell, and then released into the extracellular space. Therefore, exosomes have a stable structure and can encapsulate a wide variety of contents. Although the composition of exosomes varies among different cell types, they are mainly composed of proteins, nucleic acids, and lipids, and participate in processes such as antigen presentation, membrane transport and fusion, and immune defense (50). Lipids are a key component of the exosome membrane, ensuring the stability of the exosome membrane structure, allowing exosomes to be used as biomarkers and drug delivery vehicles (51). Delivering functional siRNA and anticancer drugs together via exosomes is an effective approach to inhibit tumor proliferation. SALL4 is overexpressed in gastric adenocarcinoma and positively correlated with tumor progression and poor prognosis. SALL4 can regulate VEGF expression by targeting the SALL4/VEGF pathway, thereby inhibiting cancer angiogenesis (52). There is increasing evidence that extracellular vesicles from mesenchymal stem cells (MSC-EXs) can have a dual role in certain cancers. Maryam Dolatshahi propose potential enhancement and combination strategies, including using MSC-EXs from foreskin and umbilical cord as sources of extracellular vesicles to improve the efficacy of gastric cancer treatment (53).

## 5 Conclusion

This study conducted a bibliometric analysis of the field of gastric cancer exosomes, and visualized some of the content to gain an overall understanding of the research hotspots and frontiers in this field. China has always been the center of this field, with high citation rates and publications, indicating that Chinese research institutions and researchers have great potential in exploring the pathogenesis of gastric cancer exosomes. Over the past 25 years, researchers have begun to focus on the unique characteristics of exosomes to explore their potential roles in gastric cancer drug therapy. The focus of research in this field has shifted from the production and function of exosomes to their roles in tumor progression and drug therapy. Based on the findings of this study, we predict that future research in this field will revolve around the mechanisms of exosomes in the pathogenesis of gastric cancer, anti-angiogenesis, tumor treatment resistance, and drug development. Researchers have started to pay attention to the unique characteristics of exosomes, so we have good reasons to believe that this field will witness more research interest and development prospects. We conducted co-citation analysis, timeline analysis, and burst detection on the references. In recent years, the key focus of gastric cancer exosomes can be summarized as the role of exosomes in drug delivery for gastric cancer, the relationship between exosomes and anti-angiogenic drugs, the relationship between exosomes and immunosuppressants, and the relationship between exosomes and drug resistance in anti-tumor drugs.

## Data Availability

The original contributions presented in the study are included in the article/**Supplementary Material**. Further inquiries can be directed to the corresponding author.
