# Supplementary material for: Research trends and hotspots in gastric carcinoma associated exosome: a bibliometric analysis
Source: Front Oncol. 2024 Dec 5;14:1457346. doi: 10.3389/fonc.2024.1457346 (PMC11655325; doi:10.3389/fonc.2024.1457346)
Supplement: Supplementary file 1 [file Table1.docx]

Supplementary Material

## Supplementary Tables

TABLE 1 Summarizes the top 5 countries with the highest volume of publications.

| Rank | Country | Documents | Citations | Average citation per paper |
| --- | --- | --- | --- | --- |
| 1 | China | 495 | 19985 | 40.37 |
| 2 | USA | 65 | 3734 | 57.45 |
| 3 | Japan | 62 | 2473 | 39.89 |
| 4 | Iran | 30 | 1080 | 36.00 |
| 5 | Singapore | 10 | 440 | 44.00 |

TABLE 2 Summarizes the top 5 institutions in terms of centrality.

| Number | Organization | Documents | Citations | Total link strength |
| --- | --- | --- | --- | --- |
| 1 | Nanjing Medical University | 41 | 2486 | 31 |
| 2 | Jiangsu University | 45 | 2695 | 21 |
| 3 | Soochow University | 20 | 863 | 16 |
| 4 | Nanjing University | 10 | 758 | 11 |
| 5 | Nantong University | 15 | 188 | 11 |

TABLE 3 Summarizes the top 5 journals in terms of centrality.

| Rank | Source | Publications | Citations | Average Citation/Publication | Country | CR | IF | h-index |
| --- | --- | --- | --- | --- | --- | --- | --- | --- |
| 1 | molecular cancer | 21 | 3812 | 181.52 | England | Q1 | 27.7 | 103 |
| 2 | cell death & disease | 12 | 692 | 57.67 | England | Q1 | 7.964 | 85 |
| 3 | cancers | 26 | 543 | 20.88 | Switzerland | Q2 | 4.9 | 53 |
| 4 | international journal of molecular sciences | 18 | 288 | 16.00 | USA | Q2 | 4.9 | 114 |
| 5 | frontiers in oncology | 38 | 484 | 12.74 | Switzerland | Q2 | 3.5 | 60 |

TABLE 4 Summarizes the top 5 leading authors with the highest publication volume.

| Rank | Author | Documents | Citations | Average Citation/Publication |
| --- | --- | --- | --- | --- |
| 1 | Ba yi | 16 | 1981 | 130 |
| 2 | Zhang haiyang | 15 | 1942 | 125 |
| 3 | Deng ting | 15 | 1943 | 124 |
| 4 | Bai ming | 13 | 1853 | 115 |
| 5 | Ning tao | 13 | 1758 | 113 |

TABLE 5 Summarizes the top 5 references in terms of centrality.

| Rank | Cited reference | Citations | Total link strength |
| --- | --- | --- | --- |
| 1 | valadi h, 2007, nat cell biol | 124 | 187 |
| 2 | jemal a, 2011, ca-cancer j clin | 129 | 161 |
| 3 | hoshino a, 2015, nature | 76 | 158 |
| 4 | pan l, 2017, j cancer res clin | 57 | 151 |
| 5 | li q, 2015, tumor biol | 55 | 129 |
